# Supplementary material for: Novel biomass-derived smoke-like carbon as a supercapacitor electrode material
Source: R Soc Open Sci. 2019 Jul 24;6(7):190132. doi: 10.1098/rsos.190132 (PMC6689642; doi:10.1098/rsos.190132)
Supplement: Supplementary information [file rsos190132supp1.docx]

Electronic support information

Novel Biomass-derived Smoke-like Carbon as a Supercapacitor Electrode Material

Mingxu Chu,^a^ Mingtang Li,*^a^ Zhaolian Han^a^, Jinshan cao^a^, Rui Li^a^, and Zhiqiang Cheng*^ab^

^a^Jilin Agriculature University, Changchun,Jilin China

E-mail: Limtdoc2008@163.com; [czq5974@163.com](mailto:czq5974@163.com).

^b^Tsinghua University, Beijing, China

E-mail: [czq5974@163.com](mailto:czq5974@163.com)

**Mingtang Li and Zhiqiang Cheng are Co-corresponding authors:*


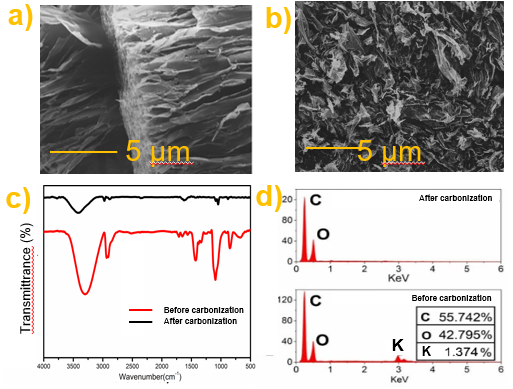


Fig. S1 (a),(b): The photograph of the raw material (polysaccharide). (c): FT-IR analysis of the polysaccharide and CPC600. (d) The edx analysis of polysaccharide and CPC600. (e), (f), (g), (h): The mapping analysis of the CPC600

As shown in Fig.S1a, the crude polysaccharide has a natural three-dimensional self-supporting mechanism. Before carbonization, in addition to the common elements in organic substances such as C, O, the polysaccharide also contains some K elements, and there are no other impurities (such as heavy metals such as Fe) that affect its electrochemical properties, and these naturally occurring activators It may also be the reason why the prepared material desired specific surface area and porosity. After carbonization, as shown in fig. S1b and d, CPCs still retains most of the original structure. As for FT-IR for the somke-like carbon (Fig S1c), the broad peak at 3500 cm^-1^ is the intermolecular and intramolecular -OH group stretching vibration peak of the fungus polysaccharide, and the double peak at 2900 cm-1 is the CH2 group in the fiber, and the peak at 1650 cm^-1^ indicates C=O. The symmetric stretching vibration, the peak at 1100 cm^-1^ is the stretching vibration of the C-O-C group. The peak at 1000 cm^-1^ indicates the presence of a pyranose ring in the polysaccharide of the fungus. After carbonization and pickling, potassium ions almost completely evaporate, so the existence of potassium is one of the key points of our further study [2].

Fig S2. The specific capacitance and coulombic efficiency of CPC600
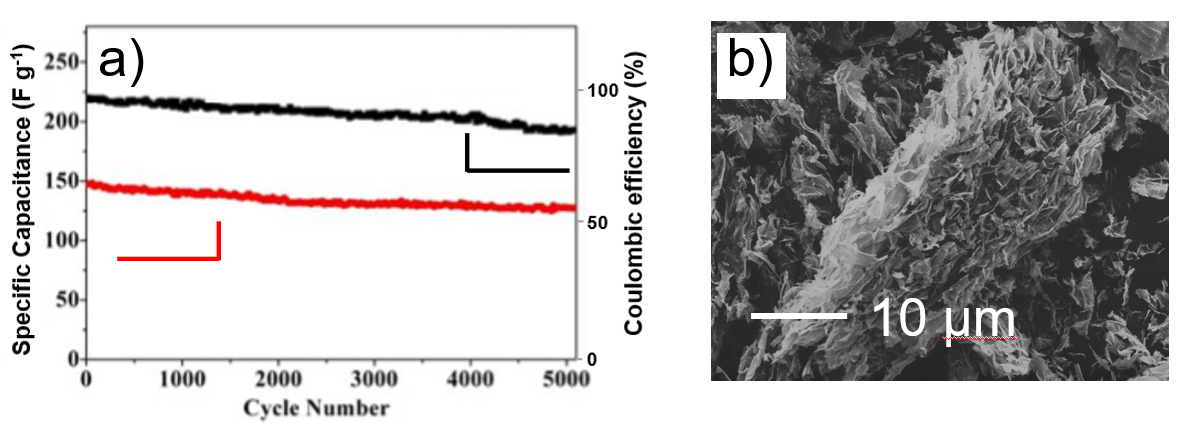


As shown in Fig S2(b), after 5000 cycles, the CPC600 has a certain agglomeration phenomenon, and its flaky structure was not obviously destroyed after thousands of cycles. Therefore, the specific capacitance of CPC600 still retains 92.1.% after 5000 cycles, as shown in fig s2(a). As for the coulombic efficiency, after 5000 cycles, the coulomb efficiency is generally stable at 90%, which indicates that CPC has good cycle stability.

1.
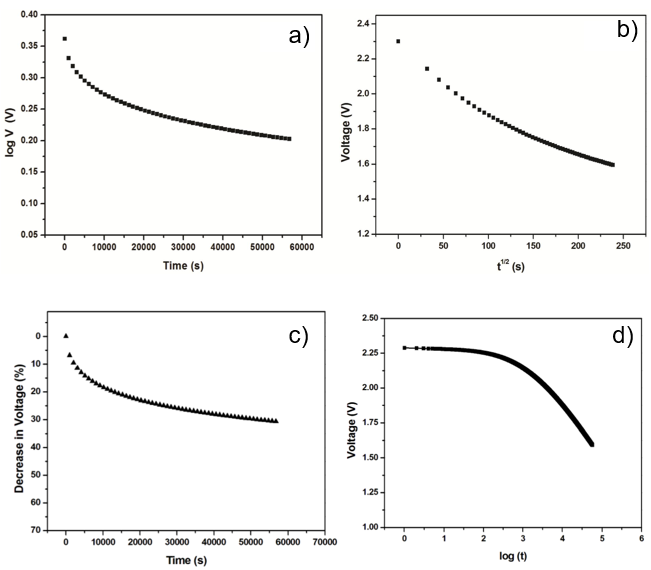
Self-discharge testing is also needed.

Fig S3. The self-discharge measurements of supercapacitors


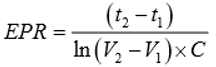
As shown in fig. S3, the self-discharge measurements of supercapacitors were carried out according to the IEC 62391-1 standard. The supercapacitors were completely discharged prior to the self- discharge study. Then, the supercapacitors were charged up to a voltage of 2.3 V with a constant current so that 95% of their rated voltage is attained within 30 minutes. It was held at rated voltage for 8 hours. After the 8 hours holding period, it was then disconnected from the voltage source. The open circuit voltage of supercapacitor was recorded using the Supercapacitor Testing System for 16 hours. The Equivalent Parallel Resistance (EPR) of supercapacitors was calculated from the open circuit voltage of the supercapacitors using equation (1):

(1)


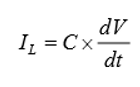
Where V1 and V2 are the voltages at time t1 and t2 respectively, C is the capacitance of supercapacitor in Farad. The leakage current of the supercapacitors was also calculated using (2)

(2)

Where IL is the leakage current, dV/dt is the slope of the curve and C is the capacitance

in Farad. Fig S3a shows the time dependent decrease in open circuit potential of graphene supercapacitors. It is obvious from fig S3 that the open circuit voltages of the supercapacitors are decreasing with respect to time. The instantaneous initial drop is due to relatively higher Equivalent Series Resistance (ESR) of graphene supercapacitors. After the instantaneous drop due to ESR, we can observe two distinct portions in the self-discharge curve of which the first one is a fast discharge portion with a shorter time duration followed by a slow discharge portion. The mechanism of self-discharge can be understood by analysing the selfdischarge curve in detail. Fig S3 (c) shows the relation between the log (self-discharge voltage) as a function of time. Generally, discharge through an ohmic leakage leads to a declining linear relation between log V and t. From Fig S2 (c), it is evident that, even after neglecting the initial drop due to ESR, the curve does not follow a perfect linear path. Hence the selfdischarge cannot be only due to the ohmic leakage pathways between the two electrodes. There can be some additional mechanism which also contributes to the self-discharge. In order to understand whether there is any diffusion-controlled mechanism, we have also plotted V vs t_1/2_. Fig S3 (d) shows the relation between self-discharge voltage decline V and t_1/2_. In general, due to diffusion controlled faradaic leakage current in carbon based supercapacitors, the open circuit voltage shows a linear declining relation with t_1/2_. As obvious from Fig S3 (d), the V and t_1/2_ have a better linear relation particularly in the slow discharge region. This clearly indicates that the diffusion-controlled mechanism will be the predominant mechanism in self discharge of these supercapacitors in addition to the ohmic leakage^[1]^. From fig S3 (c), it is clear that V vs logt curves shows no linearity. Thus it can be confirmed that there is no contribution from overcharging in the self-discharge of graphene supercapacitors. Hence it is clear that the self-discharge in these graphene supercapacitors is controlled by the combined contribution from potential controlled model due to ohmic leakage and diffusion-controlled model due to charge re-distribution phenomenon.

**1, Fabrication of electrodes and solid-state symmetric supercapacitors**

Nickel foam was first cut into rectangle sheets (20 mm * 10 mm) and treated with acetone, diluted HCl and deionized water each for 10 min ultra-sonication. A mixture containing 80 wt% active material, 10 wt% conductive carbon black and 10 wt% polyvinylidene fluoride (PVDF) was well grinded with appropriate amount of N-methyl-2-pyrrolidone (NMP) for 1 h to obtain a black paste. The paste was then casted on half of the pre-treated nickel foam and dried in a vacuum oven at 120 ºC for 8 h. The electrode was finally obtained by pressing at 10 MPa for 1 min. The loading mass of active materials on each working electrode was about 3.0 mg. The solid-state symmetric supercapacitor was assembled by two identical electrodes, wherein KOH/polyvinyl alcohol (PVA) gel was used as the electrolyte. For preparing the KOH/PVA gel, 2.0 g PVA was first mixed with 20 mL deionized water and the mixture was heated to 85 ºC under vigorous stirring until it became clear. Then, 10 mL KOH solution (6.0 M) was slowly added into the above mixture. The solution was kept stirring for 0.5 h at 85 ºC to form a clear gel electrolyte. Two electrodes were immersed in the as-prepared electrolyte for 5 min before assembly. Then, the electrodes were picked out and transferred to a fume hood at room temperature for 1 h to vaporize the excess water. Finally the electrodes were pressed together under the pressure of 1 MPa for 10 min and sealed with plastic wrap to assemble the solid-state supercapacitor. The total mass of active materials for a symmetric supercapacitor was 6.0 mg^[3]^.

**2, Fabrication of coin-type symmetric supercapacitors in ionic liquid electrolyte**

The electrochemical performances of the PGBC-based symmetric supercapacitors in ionic liquid electrolyte were measured in a two-electrode cell configuration (CR2032-type coin cell). The electrodes were prepared by coating the aforementioned mixture containing active materials onto current collectors (nickel foam) with loading mass of about 8 mg/cm2, then dried in vacuum at 120 ºC for 8 h and pressed at 10 MPa. A neat ionic liquid of 1-Ethyl-3methylimidazolium bis(trifluoromethylsulfonyl)imide (EMIM TFSI) was used as the electrolyte, and a polypropylene membrane (MPF30AC, NKK, Japan) as the separator. The coin-type supercapacitors were finally assembled in an argon-filled glove box.

Notes and references

[1] L. Shi, Li. Jin, Z, Meng, Y, C. Li and Y. Shen, *RSC Adv.* 2018, **8**, 39937 39947

[2] Y. Gong, D. L. Li, C. Z. Luo, Q. Fu, C. X. Pan, *Green Chem*. 2017, **19**, 4132 4140

[3] M. Zhou, Y. Lu, H. Chen, X. Ju, F. Xiang, *Journal of Ener stor*, 2018, **19**, 35 40

[4] K. Gao, Q. Tang, Y. Guo, L. Wang, *Journal of Elec Materi*, 2018, **47**, 337 346
